# Prevalence and risk factors of diabetes in a large community-based study in North India: results from a STEPS survey in Punjab, India

**DOI:** 10.1186/s13098-017-0207-3

**Published:** 2017-01-23

**Authors:** Jaya Prasad Tripathy, J. S. Thakur, Gursimer Jeet, Sohan Chawla, Sanjay Jain, Arnab Pal, Rajendra Prasad, Rajiv Saran

**Affiliations:** 1 International Union Against Tuberculosis and Lung Disease, The Union South East Asia Office, New Delhi, India; 2 School of Public Health, Post Graduate Institute of Medical Education and Research, Chandigarh, India; 3 Department of Internal Medicine, Post Graduate Institute of Medical Education and Research, Chandigarh, India; 4 Department of Biochemistry, Post Graduate Institute of Medical Education and Research, Chandigarh, India; 5 Department of Internal Medicine and Epidemiology, University of Michigan, Ann Arbor, MI USA

**Keywords:** Diabetes, Epidemiology, STEPS survey

## Abstract

**Aims:**

India is the diabetes capital with home to 69.1 million people with DM, the second highest number of cases after China. Recent epidemiological evidence indicates a rising DM epidemic across all classes, both affluent and the poor in India. This article reports on the prevalence of diabetes and pre-diabetes in the North Indian state of Punjab as part of a large household NCD Risk Factor Survey.

**Methods:**

A household NCD STEPS survey was done in the state of Punjab, India in a multistage stratified sample of 5127 individuals. All the subjects were administered the WHO STEPS questionnaire, anthropometric and blood pressure measurements. Every alternate respondent in the sample (n = 2499) was assayed for blood parameters.

**Results:**

Overall prevalence of DM among the study participants was found out to be 8.3% (95% CI 7.3–9.4%) whereas prevalence of prediabetes was 6.3% (5.4–7.3%). Age group (45–69 years), marital status, hypertension, obesity and family history of DM were found to be the risk factors significantly associated with DM. Out of all persons with DM, only 18% were known case of DM or on treatment, among whom only about one-third had controlled blood glucose status.

**Conclusions:**

The study reported high prevalence of diabetes, especially of undiagnosed cases amongst the adult population, most of whom have uncontrolled blood sugar levels. This indicates the need for systematic screening and awareness program to identify the undiagnosed cases in the community and offer early treatment and regular follow up.

## Background

According to International Diabetes Federation estimates, around 415 million people had DM in 2015 and this number is expected to rise to 642 million by 2040 [1]. Around 75% of subjects with DM live in low- and middle-income countries (LMICs). In financial terms, the global burden of DM is enormous, with an estimated annual expenditure of 673 billion US dollars in 2015, which constituted 12% of global health spending for that year [1]. While in urban areas of LMICs, diabetes is well recognized as a public health priority; recent prevalence data suggest that diabetes is an increasing problem among rural populations as well [2].

India is home to 69.1 million people with DM and is estimated to have the second highest number of cases of DM in the world after China in 2015 [1]. The prevalence of DM in India ranges from 5–17%, with higher levels found in the southern part of the country and in urban areas [3–9]. DM continues to increase as a result of rapid cultural and social changes, which include: ageing populations, increasing urbanization, dietary changes, reduced physical activity and unhealthy behavior. Historically a disease of the affluent, recent epidemiological evidence indicates a rising DM incidence and prevalence in urban India’s middle class and working poor [10]. Indians are also believed to have a greater degree of insulin resistance and a stronger genetic predisposition to diabetes [11].

Against this background, an understanding of the changing epidemiology of diabetes in India is required. Estimation of the prevalence of diabetes and identification of high risk groups is essential for planning of community based risk factor reduction interventions. There is currently insufficient information available on prevalence of type 2 diabetes and associated factors in North India. Previous community based studies in this region have been limited only to the Union Territory of Chandigarh which does not truly represent the North Indian population [3, 9]. Using a large cross-sectional data of 2700 individuals drawn from a multistage stratified sample in a North Indian state of Punjab, this study assesses the prevalence of type 2 DM and its associated risk factors (socio-demographic and behavioural).

## Methods

### Study design

This study reports results of a cross-sectional survey conducted in the state of Punjab, India using a multistage stratified sample.

### Study setting

Punjab is a prosperous state in northern part of India bordering Pakistan with a population of 27 million according to 2011 national census. It ranks second in terms of human development index among all states [12]. A state wide non communicable disease (NCD) risk factor survey based on WHO-STEPS approach was undertaken in Punjab in 2014–2015. The survey adopted a multistage stratified, geographically clustered sampling approach using the 2011 census sampling frame. A three-stage design was employed in urban areas whereas in rural areas a two-stage sampling design was followed. Out of a total of 100 Primary Sampling Units (PSUs), there were 60 villages from rural areas and 40 Census Enumeration Blocks from the urban locality. From each selected PSU, 54 households were selected using systematic random sampling. The ultimate sampling units were the households and one individual in the age group of 18–69 years residing in the selected household was selected using the KISH method. The details of the methodology can be found in another paper [13].

### Data collection instrument

A culturally adapted, *Punjabi* (local language) translated and pre-tested version of the WHO STEP Surveillance (STEPS) questionnaire (version 3.1) was used with minor adaptations [14]. As part of the household survey, socio demographic and behavioural information on tobacco and alcohol use, diet, physical activity, history of chronic diseases, family history of chronic conditions, health screening, and health care costs were collected in Step 1. Physical measurements such as height, weight, blood pressure and waist circumference were collected in Step 2. Biochemical tests were conducted to measure fasting blood glucose, total cholesterol, triglycerides, HDL and LDL cholesterol in Step 3.

### Data collection

Data were collected by trained investigators. SECA adult portable stadiometer was used to measure height after removing shoes, socks, slippers and any head gear. It was measured in centimetres up to 0.1 cm. SECA digital weighing scale was used to measure weight of the individuals. The scale was regularly calibrated against a standard weight. The participants were asked to remove footwear and socks and weight was recorded in kilograms up to 0.1 kg. Waist circumference was measured using a SECA constant tension tape to the nearest 0.1 cm at the level of the midpoint between the inferior margin of the last rib and the iliac crest in the mid-axillary plane. The measurement was taken at the end of a normal expiration with the arms relaxed at the sides.

One serving of vegetable was considered to be one cup of raw green leafy vegetables or 1/2 cup of other vegetables (cooked or chopped raw). One serving of fruit was considered to be one medium size piece of apple, banana or orange, 1/2 cup of chopped, canned fruit or 1/2 cup of fruit juice.

Physical activity was assessed using the Global Physical Activity Questionnaire (GPAQ), which has been developed by the World Health Organization and used in the STEPS questionnaire. This questionnaire assesses physical activity behaviour in three different domains: work, transport and during leisure time. Activities are classified into three intensity levels: vigorous, moderate and light. ‘Vigorous-intensity activities’ are activities that require hard physical effort and cause large increases in breathing or heart rate, ‘moderate-intensity activities’ are activities that require moderate physical effort and cause small increases in breathing or heart rate. Participants were classified as sufficiently active who exceed the minimum duration of physical activity per week recommended by WHO i.e. 150 min of moderate intensity physical activity or 75 min of vigorous intensity physical activity or an equivalent combination of moderate- and vigorous-intensity physical activity achieving at least 600 MET-minutes per week with each activity performed in bouts of at least 10-min duration [15]. Obesity was defined as BMI ≥ 27.5. Show cards (pictorial, adapted to the local context) were used to explain to the participants the type of physical activity, servings of fruits and vegetables and salty food intake.

Biochemical measurements (step 3): Every alternate individual (50%) of the initial sample was subjected to steps 3 (around 2700). A mix of dry and wet chemistry methods was used to assess biochemical profile of participants using blood and urine samples. For blood glucose, dry chemistry method was used by blood glucose measurement device (Optium H, Freestyle). For lipid profile i.e. cholesterol and triglycerides measurements, blood samples were drawn on individuals after 10–12 h fasting. 5 ml of venous blood was taken in sitting position, and centrifuged immediately to separate serum, and transferred under cold chain condition to the Central Reference Laboratory of Department of Biochemistry, Post Graduate Institute of Medical Education and Research, Chandigarh, India.

Laboratory measurement of triglyceride was made on Modular P 800 autoanalyzer (Roche Diagnostics, Germany) using commercially available kits (Roche Diagnostics, Germany).

### Sample size

Sample size of 4609 was calculated using the estimated prevalence of physical activity as 50%, 0.05 margin of error and 95% confidence interval. Assuming a response rate of 85%, sample size was raised to 5400. Every 2nd individual was subjected to Step 3 i.e. biochemical assessment. Out of 2700 respondents, 2499 (93%) gave consent to blood sampling for biochemical assessment.

### Data analysis

Categorical variables are summarized using proportions and continuous variables using mean or median, whichever is applicable, with 95% confidence intervals. Chi square test was used for comparison of proportions across groups and ANOVA test for comparison of means across groups. Univariate and multiple logistic regression analysis (backward conditional method) was done to determine the predictors of diabetes. Variables entered into the multiple regression models were selected on the basis of significance (p < 0.2) in the univariate analysis. Statistical analysis was done using SPSS version 16.0.

The Institute Ethics Committee of Post Graduate Institute of Medical Education and Research, Chandigarh approved the study (reference number **P-727,** dated July 21, 2014). Informed written consent was taken from all participants.

### Operational definitions

#### Dyslipidemia

National Cholesterol Education Programme (NCEP) guidelines [10] were used for definition of dyslipidemia as follows:

*Hypertriglyceridemia*—serum triglyceride levels ≥150 mg/dl (≥1.7 mmol/l).

#### Diabetes

Individuals diagnosed by a physician and/or on antidiabetic medications and/or those who had fasting blood glucose ≥126 mg/dl (≥7 mmol/l) [16].

#### Prediabetes or impaired fasting glucose [IFG]

Two different cut-offs have been used: Fasting blood glucose ≥100 mg/dl (≥5.6 mmol/l) and <126 mg/dl (<7 mmol/l) [16] and fasting blood glucose ≥110 mg/dl (≥6.1 mmol/l) and <126 mg/dl (<7 mmol/l) [17].

#### Hypertension

Individuals diagnosed by a physician and on antihypertensive medications (self-reported) and/or those who had systolic blood pressure ≥140 mmHg and/or diastolic blood pressure ≥90 mmHg—Joint National Committee 8 (JNC8) Criteria [18].

#### Current smoking

Smoking in the last one month.

Good glycemic control: defined as fasting blood glucose <130 mg/dl [16].

## Results

### Sociodemographic and behavioural characteristics

Table 1 shows the socio-demographic, behavioural and clinical characteristics of the respondents in the study. Majority of the respondents are females (64%), adults in the age group 25–44 years (51%), rural residents (61%) and belong to the general caste (48%). The prevalence of hypertension among the respondents was 36%. Nearly 15% were current alcohol users whereas around 4% were found to be current smokers (Table 1).

**Table 1:** Socio-demographic and behavioural characteristics of the study population, STEPS survey, Punjab, India, 2014–15

Characteristics	N = 2465 (%)
Age group (years)	
18–24	439 (18)
25–44	1263 (51)
45–69	763 (31)
Gender	
Male	890 (36)
Female	1575 (64)
Residence	
Rural	1492 (61)
Urban	973 (39)
Social group	
SC	926 (38)
Other backward caste	337 (14)
General	1163 (48)
Educational status	
Illiterate	575 (23)
Up to primary education	575 (23)
Up to secondary education	365 (15)
Higher education	950 (39)
Marital status	
Never married	420 (17)
Currently married	1834 (75)
Separated/divorced	31 (1)
Widowed and cohabitating	162 (7)
Current smoking	
Yes	104 (4)
No	2361 (96)
Current alcohol use^a^	
Yes	381 (15)
No	2084 (85)
Hypertension^b^	
Yes	876 (36)
No	1589 (64)

^a^One who has drank alcohol in the past 30 days

^b^Systolic Blood Pressure ≥ 140 and/or Diastolic Blood Pressure ≥ 90 or currently on medication

Figures in parenthesis indicate percentages; *SC* scheduled caste, SCs are groups of historically disadvantaged people in India recognised by the Constitution of India. Other backward caste is a collective term used by the Constitution of India to classify castes which are socially and educationally disadvantaged although they are considered to be at a less disadvantageous position than SCs and STs; General category is a term used in India to denote a group other than OBCs and SCs and are considered socially, educationally, and economically advanced

### Prevalence of DM

Overall prevalence of DM among the study participants was found out to be 8.3% (95% CI 7.3–9.4%) which was higher in urban areas (9.4%, 95% CI 7.7–11.4%) compared to rural (7.6, 6.4–9.1%), though not significant. The prevalence of prediabetes was 6.3% (5.4–7.3%) based on ADA criteria and 6.0% (5.0–7.1%) based on NPCDCS guidelines, again with a higher proportion among the urban dwellers (Table 2). On univariate analysis, the prevalence of DM was found to be significantly higher among those aged 45–69 years (18.0%), hypertensives (14.3%), obese (14.4%), with family history of DM (11.9%) and those with hypertriglyceridemia. No difference was found in prevalence by sex, residence, social group, educational status, smoking and alcohol use (Table 3).

**Table 2:** Prevalence of diabetes and pre-diabetes in Punjab, India stratified by age group, sex, and type of residence, 2014–2015

Characteristics	Prevalence of pre-diabetes N (%, 95% CI)	Prevalence of diabetes N (%, 95% CI)
Age group (years)		
18–24	12 (2.8, 1.6–4.8)	6 (1.4, 0.6–3.0)
25–44	67 (5.2, 4.1–6.5)	61 (4.7, 3.7–6.0)
45–69	77 (9.9, 8.0–12.2)	140 (18.0, 15.5–20.9)
Sex		
Male	59 (6.5, 5.1–8.3)	76 (8.4, 6.7–10.3)
Female	97 (6.1, 5.0–7.4)	131 (8.2, 7.0–9.7)
Type of residence		
Rural	88 (5.8, 4.7–7.1)	116 (7.6, 6.4–9.1)
Urban	68 (7.0, 5.6–8.8)	91 (9.4, 7.7–11.4)
Overall	156 (6.3, 5.4–7.3)^a^	207 (8.3, 7.3–9.4)

Diabetes is defined as individuals diagnosed by a physician and/or on antidiabetic medications and/or those who had fasting blood glucose ≥126 mg/dl (≥7 mmol/l); prediabetes is defined as individuals who had fasting blood glucose ≥ 100 mg/dl (≥5.6 mmol/l) and <126 mg/dl (<7 mmol/l)

^a^Prevalence of pre-diabetes based on NPCDCS guidelines- fasting blood glucose ≥ 110 mg/dl (≥6.1 mmol/l) and < 126 mg/dl (<7 mmol/l) is (6.0, 5.0–7.1) the figures in the parentheses are expressed as percentages with 95% confidence intervals

**Table 3:** Socio-economic, behavioural and clinical correlates of patients with diabetes, STEPS survey, Punjab, India, 2014–15

Characteristics	Total	Diabetes	p value	Odds ratio	95% CI	p value
Age group (years)			0.001			
18–24	430	6 (1.4)		Ref		
25–44	1289	61 (4.7)		1.5	(0.6–3.8)	0.425
45–69	776	140 (18.0)		4.7	(1.8–12.4)	0.001
Gender			0.9			
Male	907	76 (8.4)		–	–	–
Female	1588	131 (8.2)		–	–	–
Residence			0.11			
Rural	1529	116 (7.6)		Ref		
Urban	966	91 (9.4)		1.1	(0.8–1.6)	0.4
Social group			0.053			
SC	954	61 (6.4)		Ref		
Other backward caste	342	35 (10.2)		1.3	(0.8–2.1)	0.3
General	1155	107 (9.3)		1.1	(0.8–1.6)	0.6
Educational status			0.07			
Illiterate	575	51 (8.9)		Ref		
Up to primary education	599	63 (10.3)		1.3	(0.8–1.9)	0.2
Up to secondary education	1065	74 (6.9)		1.0	(0.6–1.5)	0.9
Higher education	256	19 (7.4)		1.0	(0.6–2.0)	0.9
Marital status			0.001			
Never married	413	5 (1.2)		Ref		
Currently married	1869	172 (9.2)		2.9	(1.0–8.1)	0.04
Separated/divorced/widowed	195	29 (14.9)		3.3	(1.2–10.0)	0.03
Current smoking			0.7			
Yes	101	8 (7.9)		–	–	–
No	2394	199 (8.3)		–	–	–
Current alcohol use^a^			0.109			
Yes	386	40 (10.4)		–	–	–
No	2109	167 (7.9)		–	–	–
Hypertension^b^			0.001			
Yes	1031	147 (14.3)		2.0	(1.4–2.8)	0.001
No	1464	60 (4.1)		Ref		
≥5 servings of fruits and vegetables daily^c^			0.4			
Yes	109	10 (9.2)		–	–	–
No	2386	197 (8.3)		–	–	–
Obesity (Asian cut off)^d^			0.001			
Yes	1830	96 (14.4)		1.6	(1.2–2.0)	0.03
No	665	111 (6.1)		Ref		
Family history of diabetes			0.001			
Yes	537	64 (11.9)		1.4	(1.2–1.7)	0.005
No	1925	142 (7.4)		Ref		
Family history of raised cholesterol			0.880			
Yes	126	11 (8.7)		–	–	–
No	2336	195 (8.3)		–	–	–
Family history of high blood pressure			0.965			
Yes	852	71 (8.3)		–	–	–
No	1610	134 (8.4)		–	–	–
Abdominal obesity^e^			0.001			
Yes	1415	165 (11.7)		1.5	(1.1–2.1)	0.01
No	1080	42 (3.9)		Ref		
Hypertriglyceridemia^f^			0.006			
Yes	656	136 (7.4)		0.8	(0.6–1.1)	0.2
No	1839	71 (10.8)		Ref		
Physical activity^g^						
Above minimal recommended	140	04 (3.0)	0.008	Ref		
Below minimal recommended	2355	203 (8.6)		1.8	1.5–2.1	0.01

*CI* confidence interval, *SC* scheduled caste

^a^One who has drank alcohol in the past 30 days

^b^SBP ≥ 140 and/or DBP ≥ 90 or currently on medication

^c^One serving of vegetable was considered to be one cup of raw green leafy vegetables or 1/2 cup of other vegetables (cooked or chopped raw). One serving of fruit was considered to be one medium size piece of apple, banana or orange, 1/2 cup of chopped, canned fruit or 1/2 cup of fruit juice

^d^Body mass index ≥ 27.5 kg/m^2^

^e^≥90 cm for males and ≥80 cm for females

^f^Serum triglyceride >150 mg/dl

^g^Minimum duration of physical activity per week recommended by WHO as 150 min of moderate intensity physical activity or 75 min of vigorous intensity physical activity or an equivalent combination of moderate- and vigorous-intensity physical activity achieving at least 600 MET-minutes per week with each activity performed in bouts of at least 10-min duration; Backward conditional multivariate logistic regression was performed and values are presented as odds ratio with 95% confidence interval and p value

### Risk factors for DM

Age group (45–69 years), marital status, hypertension, obesity and family history of DM were found to be the risk factors significantly associated with DM in a multivariate regression model (Table 3).

### Treatment and control status of DM

Among all persons with DM, only 37 (18%) were known case of DM or on treatment whereas the rest were newly diagnosed. Among those already on treatment or known cases of DM, 13 (35%) had controlled blood glucose status (Table 4).

**Table 4:** Treatment and control status among diabetic patients in Punjab, STEPS survey, 2014–15

Demographic variables	Total diabetics N	On treatment N (%)	Good glycemic control N (%)
Total	N = 207	N = 37 (18)	N=13 (35)
Gender			
Male	76	16 (21)	6 (38)
Female	131	21 (16)	7 (33)
Age (in years)			
18–24	6	2 (33)	2 (100)
25–44	61	12 (20)	4 (33)
45–69	140	23 (16)	7 (30)
Residence			
Rural	116	22 (19)	9 (41)
Urban	91	15 (16)	4 (27)

Good glycemic control is defined as fasting blood glucose <130 mg/dl

## Discussion

Overall prevalence of DM and prediabetes among the study participants was 8.3 and 6.3% respectively. Only 18% of all cases of DM were already known case or on treatment, among whom only about one-third had controlled blood glucose status.

This state-wide study was done in one of the most prosperous states in India. There have been few large community based studies looking at prevalence of diabetes in India. The National Urban Diabetes Survey showed an age-standardised prevalence of 12.1% for diabetes and 14% for IGT in six large metropolitan cities [5]. The Prevalence of Diabetes in India Study (PODIS) reported lower diabetes prevalence of 5.9 and 2.7% in urban and rural areas respectively with an overall prevalence of 4.3% [19]. Two studies in Chandigarh, a very prosperous city in North India, showed high prevalence of diabetes. In the INDIAB study, the city was found to have the highest prevalence of diabetes (13.6%) [3]. The Chandigarh Urban Diabetes Survey (CUDS) also reported high prevalence of diabetes and prediabetes i.e. 11.1 and 13.2% respectively [9].

Barik et al. in a large cross-sectional survey in rural West Bengal, which is situated in the eastern region of the country, found that the prevalence of diabetes and pre-diabetes among adults >18 years was 2.95 and 3.34% respectively [6]. In another study, Little et al. reported a high prevalence of type 2 diabetes (10.8%) among adults population (>19 years) in rural parts of South India [4]. These figures imply that though the prevalence of DM varies in different settings, it is certainly quite high and warrants immediate attention.

Our study adds to the limited but growing body of evidence suggesting that diabetes is no longer confined to urban areas of India and is a matter of concern in rural areas as well [4]. Considering the fact that over 70% of the population of India are rural and often faced with issues like poverty, poor access to health care, this is quite a worrisome finding. The present study reported no gender difference in the prevalence of DM which is supported by evidence from other studies in India [5, 6, 20], although a few studies have shown a male preponderance [3, 21].

Generalized obesity/high BMI and abdominal obesity were independently associated with diabetes which is similar to the results in most other studies [3–6, 9, 21]. Indians have a lower BMI than those of European descent. However, the risk of diabetes increases at very low levels of BMI for Indians [22]. Poor physical activity was also associated with diabetes as supported by earlier studies [4, 6]. The protective effects of physical activity against obesity, cardiovascular disease, and metabolic syndrome have already been proven [23]. The associations remained even after controlling for anthropometric measures, indicating that physical activity may have a direct impact on risk of diabetes apart from its association through obesity [4, 24]. Efforts that focus on healthy diet and promoting physical activity have the potential to reduce the risk of obesity, the single most important risk factor for type 2 diabetes [6]. Family history of DM is a strong predictor of the disease which is supported by most other studies [3, 5, 9]. The study results indicate that elderly individuals, hypertensives, obese (general/abdominal) or those with a family history of DM constitute an important group for screening.

Nearly 80% of individuals with diabetes were previously undiagnosed. The ratio of undiagnosed to total patients with DM was much higher compared to another study in rural Tamil Nadu conducted by Anjana et al. (48% undiagnosed) [3]. In another study in Delhi, only one-third of the diabetic patents were aware of their condition [25] thereby indicating the need for aggressive screening programs. Despite screening for NCDs including diabetes being a major component of the National Programme for Control of Cancer, Diabetes, Cardiovascular Diseases and Stroke (NPCDCS) in India, implementation is dismal [26]. The government of India has taken certain initiatives at national level which is appreciable, but there is a need to implement it at grass root level before the disease takes the shape of a pandemic in India. The pool of undiagnosed cases of DM left untreated is more prone to microvascular as well as macrovascular complications. Hence, it is necessary to identify and offer early therapy to these individuals and ensure regular follow up. The study results show that among persons with known DM on treatment, nearly two-third had uncontrolled blood glucose levels. Further studies are required to understand the reasons for the same.

The strengths of the study include a large multistage stratified community based sample, high participant response rate, use of standardized STEPS questionnaire and robust methodology and adherence to STROBE reporting guidelines [27].

### Study limitations

The study had few limitations. Firstly, this being a cross-sectional study, prevents us from drawing causal inferences. Secondly, measurement of blood glucose was done by a glucometer device instead of venous blood glucose estimation due to logistic constraints. However, regular quality control check on blood glucose measurement was done in a reference laboratory as per the manufacturer’s instructions. Thirdly, only fasting blood glucose was used to diagnose diabetes and pre-diabetes.

## Conclusion

In conclusion, the present study provides reliable and recent epidemiological information regarding the high burden of diabetes mellitus among the adult population in a representative North Indian population. Around 15% of the general adult population have diabetes or pre-diabetes, calling for an urgent attention. This study also highlights a significant burden of undiagnosed cases of DM in the community, most of them are poorly controlled. There is need to identify the large pool of undiagnosed cases of DM and offer early treatment in order to avoid complications.

## Abbreviations

NCD: non communicable disease
DM: diabetes mellitus
LDL: low density lipoproteins
HDL: high density lipoproteins
GPAQ: Global Physical Activity Questionnaire
BMI: body mass index
